# Bacterial Translocation Associates With Aggression in Schizophrenia Inpatients

**DOI:** 10.3389/fnsys.2021.704069

**Published:** 2021-09-29

**Authors:** Chong Wang, Teng Zhang, Lei He, Ji-Yong Fu, Hong-Xin Deng, Xiao-Ling Xue, Bang-Tao Chen

**Affiliations:** ^1^Department of Psychiatry, Zhumadian Psychiatric Hospital (The Second People's Hospital of Zhumadian), Zhumadian, China; ^2^Department of Hematology, The Third Affiliated Hospital of Chongqing Medical University, Chongqing, China; ^3^Department of Dermatology, Chongqing University Three Gorges Hospital, Chongqing, China

**Keywords:** schizophrenia, aggression, bacterial translocation, inflammation, association

## Abstract

**Objective:** Accumulating evidence indicates that inflammation abnormalities may contribute to aggression behaviors in psychotic patients, however, the possible sources of inflammation remain elusive. We aimed to evaluate the associations among aggression, inflammation, and bacterial translocation (BT) in aggression-affected schizophrenia (ScZ) inpatients with 2 weeks of antipsychotics discontinuation.

**Methods:** Serum specimens collected from 112 aggression and 112 non-aggression individuals with ScZ and 56 healthy adults were used for quantifications of inflammation- or BT-related biomarkers. Aggression severity was assessed by Modified Overt Aggression Scale (MOAS).

**Results:** Proinflammation phenotype dominated and leaky gut-induced BT occurred only in cases with ScZ with a history of aggression, and the MOAS score positively related to levels of C-reactive protein, interleukin (IL)-6, IL-1β, and tumor necrosis factor-α. Furthermore, serum levels of BT-derived lipopolysaccharide (LPS), as well as LPS-responded soluble CD14, were not only positively correlated with levels of above proinflammation mediators but also the total MOAS score and subscore for aggression against objects or others.

**Conclusion:** Our results collectively demonstrate the presence of leaky gut and further correlate BT-derived LPS and soluble CD14 to onset or severity of aggression possibly by driving proinflammation response in inpatients with ScZ, which indicates that BT may be a novel anti-inflammation therapeutic target for aggression prophylaxis.

## Introduction

Schizophrenia (ScZ) is a chronic and heterogeneous psychiatric syndrome characterized by recurrent episodes of acute psychosis alternating with periods of full or partial remission. Globally, ScZ affects ~1% of the population and occurs mainly in individuals in the late adolescence or early adulthood (Kahn et al., 2015; Charlson et al., 2018). It covers a broad spectrum of clinical symptoms including positive symptoms (delusions, hallucinations, etc.), negative symptoms (anhedonia, social withdrawal, poverty of thought, etc.), and cognitive dysfunction. Current treatment modalities are available only for symptoms mitigation, thus, significant disability, insupportable psychosocial burdens, and premature mortality are of great concerns (Tiihonen et al., 2017; Stepnicki et al., 2018).

Compared with the general population, inpatients with ScZ are four to seven times more likely to commit aggression acts involving verbal threat, assault, and homicide, which brings a great challenge for both mental health services and public safety (Cho et al., 2019). Aggression is more inclined to be an independent entity. The manifold pathogenesis of aggression in ScZ is complicated by elevated serum C-reactive protein (CRP) and increased ratio of CRP to interleukin (IL)-10, which arouses increasing concerns about the role of systemic inflammation in the onset or severity of aggression in ScZ (Barzilay et al., 2016; Das et al., 2016; Zhang et al., 2017). Inflammation phenotype involves the integration of various pro-/anti-inflammatory cytokines. Interleukin-6, IL-1β, and tumor necrosis factor (TNF)-α are well-proved proinflammatory cytokines responsible for initiation and exacerbation of inflammation, and the serum levels of them were demonstrated to be significantly upregulated in patients with ScZ in most of the related studies (Lesh et al., 2018; Momtazmanesh et al., 2019). Although other cytokines such as interferon (IFN)-γ, IL-4, IL-17, IL-10, and transforming growth factor (TGF)-β were also proved to be linked with ScZ, they may promote or suppress inflammation response in the different subsets of cases with ScZ (Lesh et al., 2018; Momtazmanesh et al., 2019). Crossing blood-brain barrier, the peripheral cytokines precipitate changes in mood and behavior through hypothalamic–pituitary–adrenal axis (Petra et al., 2015; Singh et al., 2019; Misiak et al., 2020), which lays a structural foundation for studying the involvement of inflammation in aggression. However, the mentioned cytokines except serum CRP are rarely profiled and potential sources of peripheral inflammation, with exception of being overweight or lack of dental care, are seldom explored (Fond et al., 2021) in individuals with aggression (Ag)-affected ScZ (ScZ-Ag).

Interestingly, inflammation abnormalities could be caused by alterations in the gut microbiome and the recent evidence from human metabolomics suggested a correlation between enteric dysbacteriosis and dysfunction of neurochemical pathways including inflammation activation underlying aggression in patients with ScZ (Severance et al., 2016; Manchia and Fanos, 2017; Chen et al., 2021; Zeng et al., 2021). Changes in gut microbiota may compromise the integrity of the intestinal tract (leaky gut) and subsequently cause a higher translocation rate of bacterial immunogenic components such as bacterial DNA (BactDNA) and lipopolysaccharide (LPS) from gut into peripheral circulation, which in turn activate immuno-inflammatory signaling (Francés et al., 2007; Martin-Subero et al., 2016). The so-called bacterial translocation (BT) was extensively proved to be correlated with various inflammation-involved diseases and with negative symptoms or neurocognitive impairments in deficit cases with ScZ (Caso et al., 2016; Maes et al., 2019a; Severance et al., 2020). However, the occurrence of leaky gut-related BT and its association with systemic inflammation in ScZ-Ag are poorly investigated.

Taken together, we hypothesize that proinflammation cytokines characterize the aggression behaviors in patients with ScZ and increased intestinal permeability-caused BT is one of the main culprits for the tuning process of inflammation. With regard to this, we determined serum levels of aforementioned inflammation cytokines, leaky gut and BT-related biomarkers, and further assessed the correlations between BT biomarkers and inflammation cytokines or the severity of aggression, in the hope of providing more convincing evidence for BT-derived inflammatory pathogenesis of aggression in ScZ.

## Materials and Methods

### Study Population

The prospective and controlled investigation was conducted in inpatients with ScZ with or without aggression behaviors within 1 week prior to admission during November 2019 to November 2020 in the Second People's Hospital of Zhumadian, a tertiary psychiatric hospital in Henan Province, China. At sample collection, all included inpatients with ScZ were at least 2 weeks of antipsychotics discontinuation. Inpatients with ScZ with the presence of aggression behaviors within 1 week prior to admission and absence of any aggression behaviors during disease course before enrollment were classified into ScZ-Ag and NScZ-Ag groups, respectively.

For comparison, age-, gender-, and body mass index (BMI)-matched healthy volunteers recruited during the same period with no history of psychiatric or medical illness were set as control (Ctrl group) and the ratio of healthy volunteers: cases with ScZ-Ag is 1:2. All the subjects were aged ≥18 years. The diagnosis was made by two board-certified psychiatrists according to the 10th edition of the international classification of diseases (ICD-10) criteria for ScZ. Exclusion criteria included: (a) aggression behaviors not within 1 week prior to admission; (b) pregnant or lactating women; (s) presence of any other psychoses including affective disorder or substance abuse; (d) comorbidity with severe somatic diseases or neurological diseases; (e) comorbidity with other medical conditions such as parenchymal organ-specific diseases, immune-related diseases, hematological diseases, gastrointestinal diseases, and any history of gastrointestinal surgeries; (f) use of systemic corticosteroids, any other immunosuppressive therapy, and oral probiotics in the recent 3 months; (g) inpatients with fever (>37.9°C) or those who were treated with antibiotics, antipyretics or anti-inflammatory medications in the recent 2 weeks. The study was approved by and carried out under the guidelines of the Ethics Committee of the Hospital, and written informed consent was obtained from all the healthy volunteers, the inpatients or the guardians of inpatients (if the patients were unable to sign consent because of poor intelligence) at the time of recruitment.

#### Subjects Profiles

A structured questionnaire was used to collect data on general sociodemographic variables (age, gender, occupation, education background, ethnicity, height and weight, family income, living circumstance and marriage status), health status (medical history, current medications and family history), and living habits (alcohol intake and smoking pattern) in all the participants. In inpatients with ScZ, the information on specific conditions including the onset of illness and the type of aggression was inquired.

### Clinical Assessments

Modified Overt Aggression Scale (MOAS) was used to characterize aggression behaviors observed within the past 1 week. It involves four subscales and a score from zero to four is assigned for each type of aggression with zero indicating no aggression and higher scores pointing to increasing severity. The score of each subscale is then multiplied by a predefined loading (one for verbal aggression, two for aggression against objects, three for self-aggression, and four for aggression against other people) and the sum of each subscale-weighted score (range 0–40) is referred to the total score. Inpatient with a total score of zero or only having a score of one or more for verbal aggression was classified as being the non-aggressive (Huang et al., 2009). The presence and severity of each psychiatric symptom in cases with ScZ were evaluated by the positive and negative syndrome scale (PANSS) involving positive symptom subscale (seven items), negative symptom subscale (seven items), general psychopathological subscale (16 items), and supplemental items (three items). Each item on the subscale score from one to seven base on the frequency and severity of the symptom (Kelley et al., 2013).

### Blood Sampling and Laboratory Detection

Fasting peripheral blood samples were collected from all the subjects at 8:00 a.m. Blood cell count and liver function were examined routinely. The protein levels of indicators assessed by enzyme-linked immunosorbent assay (ELISA) in this study involved CRP (#E007462, 3ABio, Shanghai, China), IL-6 (#E000482, 3ABio, Shanghai, China), IL-1β (#E001772, 3ABio, Shanghai, China), IL-4 (#DG10308H, Dogesce, Beijing, China), IL-10 (#DG10495H, Dogesce, Beijing, China), IL-17 (#DG10431H, Dogesce, Beijing, China), IFN-γ (#C608-01, GenStar, Beijing, China), TNF-α (#489204, Cayman, Michigan, USA), TGF-β (#DG10113H, Dogesce, Beijing, China); leaky gut-related biomarkers[intestinal fatty acid-binding protein (I-FABP, #DFBP20, R&D Systems, Minnesota, USA) and Claudin-3 (#abx250611, Abbexa, Cambridge, UK)]; BT-related biomarkers[LPS (#DG11072H, Dogesce, Beijing, China), soluble CD14 (sCD14, #DC140, R&D Systems, Minnesota, USA), and endotoxin core antibody (EndoCAb, #E013362, 3ABio, Shanghai, China)]. Assays were performed according to the specifications of the manufacturer and the detection limits were in line with the instructions of the manufacturer. Each serum sample was measured in duplicate. All the plates were read by the I Mark™ Micro plate Reader (Bio-Rad, Hercules, California, United States).

### Quantification of BactDNA Fragments

Quantification of circulating BactDNA fragments and quality control were performed as described previously (Such et al., 2002; Ericsen et al., 2016). To avoid potentially bacterial contamination of molecular biology reagents, all the specimens were processed in airflow chambers by the same investigator and all the tubes were never exposed to free air. To remove potentially confounding 16S rDNA contamination, six tubes of prepared diethyl pyrocarbonate (DEPC) water were set as negative controls and the processes of water from DNA extraction to quantitative PCR (qPCR) were completely synchronized with those of blood.

Genomic DNA was extracted from 200 μl of serum or DEPC water using QIAmp DNA Blood Minikit (Qiagen, Hilden, Germany) according to the instructions of the manufacturer and DNA was eluted in a 100 μl final volume. BactDNA levels were determined by qPCR in an amplification reaction of 20 μl with forward primer (5′-AGAGGGTGATCGGCCACA-3′) and reverse primer (5′-TGCTGCCTCCCGTAGGAGT-3′), the universal eubacterial primers of a conserved region of 16S rDNA gene (Francés et al., 2004). The amplification conditions for the 59 base pairs of DNA fragments were 95°C for 10 min, followed by 45 cycles at 95°C for 15 s and 60°C for 60 s. Each sample was amplified in triplicate and the BactDNA content was calculated according to a standard curve that generated from serial dilutions of plasmid DNA containing known copy numbers of the template. The final circulating BactDNA concentration was calculated by subtracting the proportion of 16S rDNA copies/μl detected in water controls from those in blood.

### Statistical Analyses

Statistical analysis of the data compiled in Excel databank was conducted using SPSS/PC software (Version 19.0 for Windows; SPSS Inc., China). Categorical and continuous variables were expressed as number (%) or mean (M) ± SD, respectively. Normal distribution of raw data was inspected by Kolmogorov–Smirnov tests, and IL-17, IGF-β, and EndoCAb were logarithmically transformed to achieve Gaussian distributions. There were no outliers in MOAS score, PANSS score, cytokines, and bacterial measures by inspection of related boxplots. For comparison of demographic information and clinical characteristics at baseline among groups, Fisher's exact Chi-square test or one-way ANOVA were conducted except specification. Analysis of covariance (ANCOVA) controlling for age, gender, BMI, and course with ScZ was used to analyze cytokines and bacterial measures among the three groups, and Bonferroni's multiple comparison test that can calculate the corrected statistical significance for multiple comparisons was performed for *post-hoc* analysis of pairwise comparisons. Partial correlation analysis controlling for episodes with ScZ, course with ScZ, income levels, marriage status, education background, and occupation was used to determine the relationship between clinical symptoms and inflammation cytokines or bacterial measures. All the tests were two-sided. A *P* < 0.05 was accepted as the cutoff for statistical significance.

## Results

### Inpatients Characteristics

During the time of study, a total of 528 adult inpatients with ScZ demonstrated a history of aggression behaviors prior to hospitalization. By excluding cases with <2 weeks of antipsychotics discontinuation (56 cases), aggression occurred prior to 1 week time period preceding hospital admission (135 cases), aggression occurred prior to and within 1 week (184 cases), and aggression occurred only within 1 week but met the aforementioned exclusion criteria (41 cases), only 21.2% (112/528) of them [average total MOAS score, mean(SD), 16.4(8.2)] were included in ScZ-Ag group as defined previously. In this study, 112 age-, gender-, and BMI-matched NScZ-Ag inpatients [average total MOAS score, mean(SD), 1.6(0.9)] and 56 healthy volunteers were included. As Table 1 showed, there was statistical significance in terms of income, marriage, education level, and occupation among the three groups (*P* < 0.001 for all variables). Compared with NScZ-Ag group, more aggression inpatients were single (58.9 vs. 31.2%, *P* = 0.010) and a much higher proportion of aggression cases had low income (64.3 vs. 36.7%, *P* = 0.017) and poor education background (73.2 vs. 43.8%, *P* = 0.021). Between inpatients with ScZ with and without aggression, there was no statistical difference regarding ethnicity, living conditions, occupation distribution, episodes with ScZ, course with ScZ, and total PANSS score (*P* > 0.05 for all the variables).

**Table 1:** Clinic characteristics of all inpatients at baseline.

**Items**	**ScZ-Ag group**	**NScZ-Ag group**	**Ctrl group**	***P*-value**
Case No.	112	112	56	-
Female, n (%)	67 (59.8)	64 (57.1)	35 (62.5)	0.792
Age, mean (SD), years	33.5 (8.4)	34.2 (9.1)	33.8 (8.7)	0.835
BMI, mean (SD), kg/m^2^	21.9 (2.4)	21.7 (2.1)	22.3 (1.9)	0.243
Ethnic Han, n (%)	105 (93.8)	109 (97.3)	52 (92.9)	0.336
Low income, n (%)	72 (64.3)	41 (36.7)	17 (30.4)	**0.000**
Living with families, n (%)	91 (81.3)	96 (85.7)	45 (80.4)	0.579
**Marriage status, n (%)**				
Married	18 (16.1)	33 (29.5)	35 (62.5)	**0.000**
Single	66 (58.9)	35 (31.2)	15 (26.8)	
Divorced	26 (23.1)	37 (33.0)	6 (10.7)	
Widowed	2 (1.9)	7 (6.3)	0 (0)	
**Education background, n (%)**				
Elementary school and below	82 (73.2)	49 (43.8)	12 (21.4)	**0.000**
Middle and high school	15 (13.4)	39 (34.8)	16 (28.6)	
College and above	15 (13.4)	24 (21.5)	28 (50.0)	
**Occupation, n (%)**				
Physical labor	21 (18.8)	22 (19.7)	18 (32.1)	**0.000**
Mental labor	13 (11.6)	10 (8.9)	29 (51.8)	
Unemployment	78 (69.6)	80 (71.4)	9 (16.1)	
No. of ScZ episodes^&^	5.6 (2.7)	4.9 (3.2)	NA	0.319
ScZ course, mean (SD), years^&^	7.4 (4.3)	6.9 (4.1)	NA	0.431
Total MOAS score^&^	16.4 (8.2)	1.6 (0.9)	0 (0.0)	**0.000**
Total PANSS score^&^	65.2 (8.3)	63.4 (7.5)	0 (0.0)	0.090

*ScZ-Ag, schizophrenia with aggression; NScZ-Ag, schizophrenia without any aggression; BMI, body mass index; MOAS, Modified Overt Aggression Scale; PANSS, positive and negative syndrome scale; NA, not applicable; &, analysis using Student's t-test between ScZ-Ag and NScZ-Ag groups. The meaning of the bold values indicate P < 0.05*.

### Inflammation and Severity of Aggression

**Figure 1:**
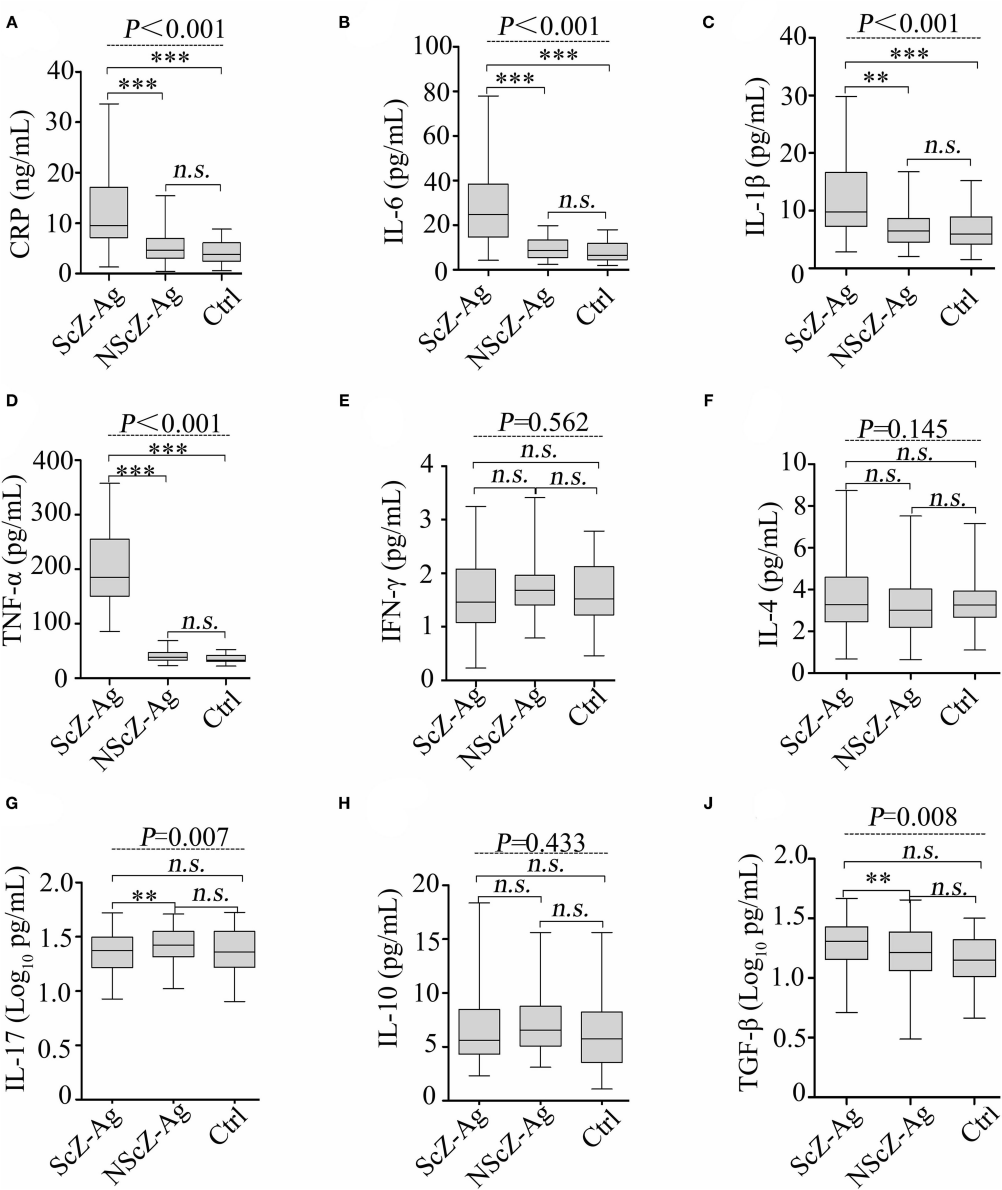
Protein levels of serum CRP **(A)**, IL-6 **(B)**, IL-1β **(C)**, TNF-α **(D)**, IFN-γ **(E)**, IL-4 **(F)**, IL-17 **(G)**, IL-10 **(H)** and TGF-β **(J)** in peripheral blood of subjects. CRP, C-reactive protein; IL, interleukin; TNF, tumor necrosis factor; TGF, transforming growth factor. Data were presented as boxplots. In *post-hoc* analysis using Bonferroni's multiple comparison test, *n.s*. > 0.05, *P < 0.05, **P < 0.01, ***P < 0.001 analysis using ANCOVA.

**Figure 2:**
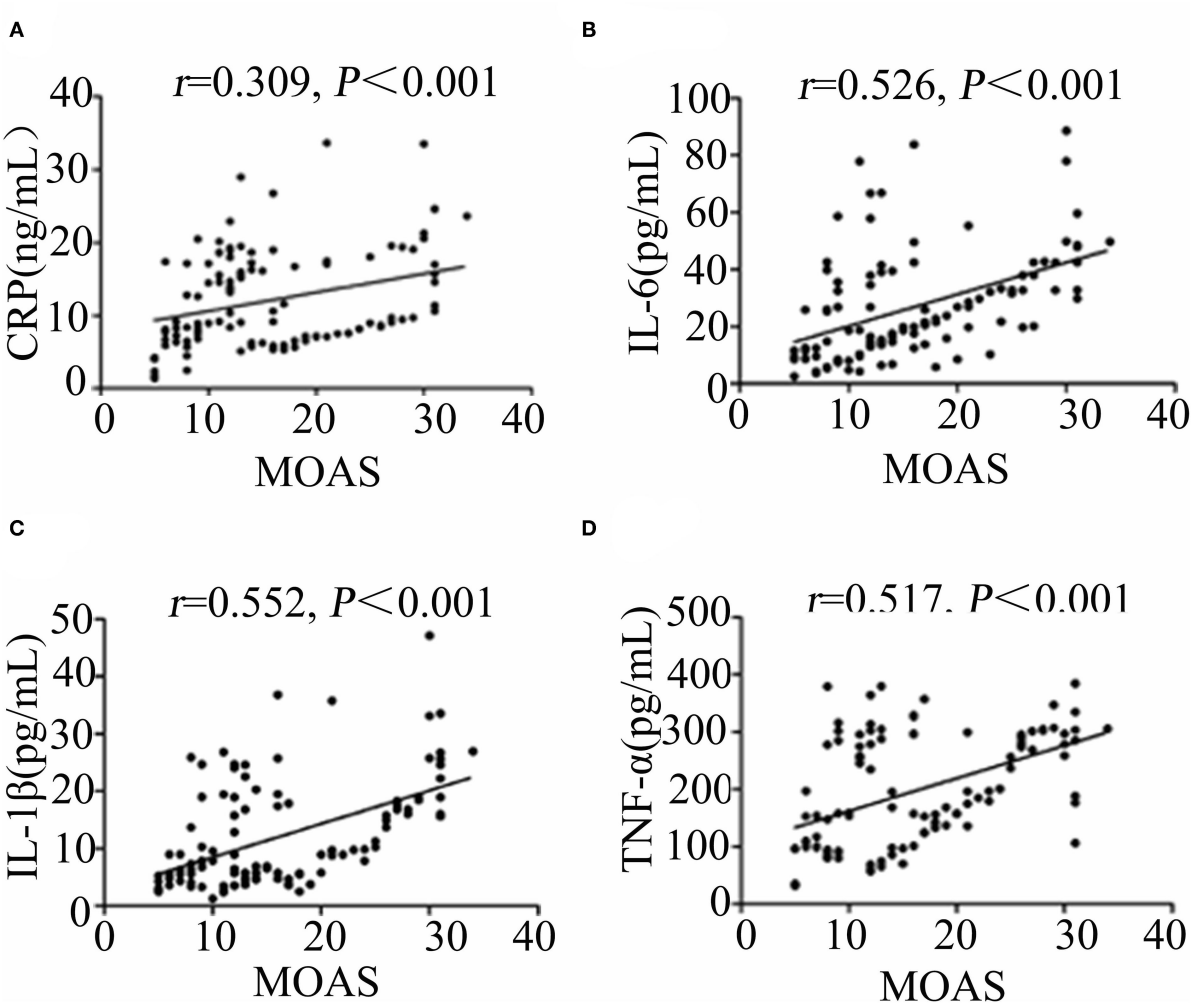
Partial correlation analyses of MOAS with CRP **(A)**, IL-6 **(B)**, IL-1β **(C)**, and TNF-α **(D)** in ScZ-Ag group.

As shown in Figure 1, the results of ANCOVA analysis displayed that there were statistically significant differences between ScZ-Ag, NScZ-Ag, and Ctrl groups in terms of CRP (*F* = 75.2, *P* < 0.001), IL-6 (*F* = 102.00, *P* < 0.001), IL-1β (*F* = 37.90, *P* < 0.001), TNF-α (*F* = 450.00, *P* < 0.001), IL-17 (*F* = 7.00, *P* = 0.007), and TGF-β (*F* = 7.55, *P* = 0.008). Further, *post-hoc* analysis using Bonferroni's multiple comparison test found that none of inflammatory markers differed significantly between NScZ-Ag and Ctrl groups (all *P* > 0.05), while serum levels of CRP, IL-6, IL-1β, and TNF-α dramatically increased approximately two to five times on average in ScZ-Ag group in comparison with NScZ-Ag group (all *P* < 0.001). On partial correlation analysis controlling potential confounders, serum levels of CRP (*r* = 0.309, *P* < 0.001), IL-6 (*r* = 0.526, *P* < 0.001), IL-1β (*r* = 0.552, *P* < 0.001), and TNF-α (*r* = 0.517, *P* < 0.001) were all positively associated with total MOAS score in ScZ-Ag group (Figure 2). Altogether, these results indicate that systemic proinflammation response mainly occurs in inpatients with ScZ with aggression behaviors.

### BT Determination and Its Association With Inflammation

**Figure 3:**
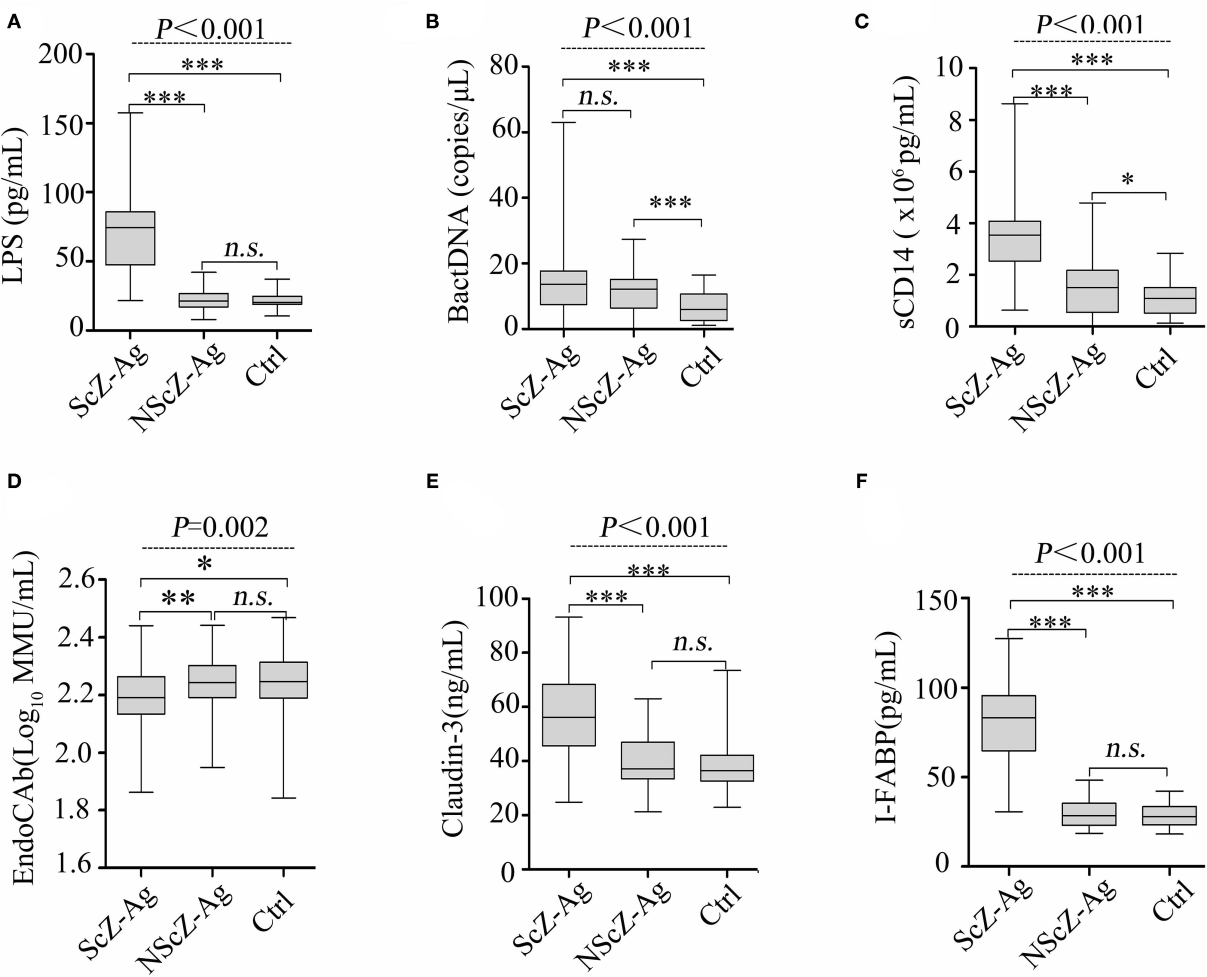
Serum levels of LPS **(A)**, BactDNA **(B)**, sCD14 **(C)**, EndoCAb **(D)**, Claudin-3 **(E)** and I-FABP **(F)** among the three groups. LPS, lipopolysaccharide; BactDNA, bacterial DNA; sCD14, soluble CD14; EndoCAb, endotoxin core antibody; I-FABP, intestinal fatty acid-binding protein. Data were presented as boxplots. In *post-hoc* analysis using Bonferroni's multiple comparison test, *n.s*. > 0.05, *P < 0.05, ***P < 0.001 analysis using ANCOVA.

To explore the source of proinflammation phenotype, BT-related serum biomarkers in all the subjects were measured (Figure 3). Regarding biomarkers of “leaky gut” (Claudin-3 and I-FABP), bacterial components (LPS and BactDNA), and LPS-response products (sCD14 and EndoCAb), statistically significant differences between the three groups were observed (all *P* < 0.01) from ANCOVA analysis results. *Post-hoc* analysis showed that only BactDNA titers (11.79 ± 6.97 vs. 7.19 ± 4.76 copies/μl, *P* < 0.001) and sCD14 levels (1.57 ± 1.15 vs. 1.07 ± 0.61 × 10^6^ pg/ml, *P* < 0.05) were moderately increased in NScZ-Ag group than Ctrl group, while serum concentrations of Claudin-3 (58.47 ± 13.52 vs. 39.27 ± 9.61 ng/ml, *P* < 0.001), I-FABP (80.47 ± 21.47 vs. 29.56 ± 7.46 pg/ml, *P* < 0.001), LPS (73.51 ± 32.29 vs. 23.16 ± 7.83 pg/ml, *P* < 0.001), sCD14 (3.45 ± 1.39 vs. 1.57 ± 1.15 × 10^6^ pg/ml, *P* < 0.001) were significantly higher, EndoCAb concentration (2.18 ± 0.13 vs. 2.23 ± 0.11 log_10_ MMU/ml, *P* < 0.01) was remarkably lower in ScZ-Ag group than NScZ-Ag group. In ScZ-Ag group (Table 2), circulating concentration of LPS was further found to be positively correlated with CRP (*P* < 0.001), IL-1β (*P* = 0.001) and TNF-α (*P* = 0.006), sCD14 was positively associated with CRP (*P* < 0.001), IL-6 (*P* = 0.007), and TNF-α (*P* = 0.040) after controlling potential confounders. These data not only indicate the presence of “leaky gut,” but also imply the link that circulating LPS from BT, as well as LPS responded sCD14, might be the important cause synergistically leading to the higher levels of proinflammation mediators observed in inpatients with ScZ with any type of aggression behaviors.

**Table 2:** Relations of bacterial translocation markers to cytokines in ScZ-Ag group.

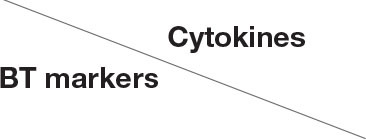	**CRP**		**IL-6**		**IL-1β**		**TNF-α**	
	***r***	***P***	***r***	***P***	***r***	***P***	***r***	***P***
LPS	0.713	0.000^*^	0.212	0.298	0.627	0.001^*^	0.583	0.006^*^
BactDNA	0.201	0.329	0.196	0.472	0.098	0.592	0.302	0.129
sCD14	0.826	0.000^*^	0.509	0.007^*^	0.056	0.613	0.341	0.040^*^
EndoCAb	−0.239	0.269	−0.117	0.523	−0.049	0.617	−0.316	0.084
I-FABP	0.112	0.564	0.082	0.613	0.067	0.627	0.298	0.158
Claudin-3	0.257	0.218	0.286	0.182	0.318	0.065	0.125	0.499

* *P < 0.05. Analyses using partial correlation analysis. The meaning of the bold values indicate P < 0.05*.

### Correlation of BT With Symptoms Dimensions

Partial correlation analyses in inpatients with aggression (Table 3) showed that total MOAS score was positively associated with protein levels of circulating LPS (*r* = 0.412, *P* = 0.005) or sCD14 (*r* = 0.267, *P* = 0.035). Regarding the subscale of MOAS, only aggression against objects (*r* = 0.406, *P* = 0.006) or toward others (*r* = 0.326, *P* = 0.011) were found to be correlated positively with circulating LPS, and such associations with the circulating sCD14 were also detected. In addition, results showed statistically significant association between positive PANSS and circulating LPS (*r* = 0.298, *P* = 0.023) or sCD14 (*r* = 0.315, *P* = 0.015). Altogether, the data further suggest that the increased protein levels of LPS or sCD14 in peripheral blood potentially initiate aggression behaviors in inpatients with ScZ *via* exacerbating the severity of systemic inflammation.

**Table 3:** Correlations of Lipopolysaccharide or sCD14 with severity aggression.

**Items**	**LPS**		**sCD14**	
	***r***	***P***	***r***	***P***
Total MOAS score	0.412	0.005^*^	0.267	0.035^*^
Verbal aggression	0.198	0.263	0.154	0.299
Aggression against objects	0.406	0.006^*^	0.397	0.008^*^
Self-aggression	−0.054	0.512	−0.067	0.476
Aggression toward others	0.326	0.011^*^	0.256	0.042^*^
Total PANSS score	0.068	0.477	0.118	0.364
Positive	0.298	0.023^*^	0.315	0.015^*^
Negative	−0.136	0.317	−0.098	0.418
General	0.049	0.574	0.009	0.832

* *P < 0.05. Analyses using partial correlation analysis. The meaning of the bold values indicate P < 0.05*.

## Discussion

Aggression can attack individuals with or without psychosis. It is one of the top 20 causes of disabilities worldwide that is present in 15.3–53.2% of inpatients with ScZ in China (Zhou et al., 2016). Growing evidence demonstrate that the serious public health problem is the resultant of pro-/anti-inflammation imbalance, since some inflammation cytokines were proved to be involved in the pathogenesis of ScZ (Müller et al., 2015; Petrikis et al., 2015; Momtazmanesh et al., 2019; Feng et al., 2020; Park and Miller, 2020). However, the role of and alterations in these cytokines may be variable in different stratifications of ScZ, antipsychotic drugs used or presence of aggression behaviors is a case (Petrikis et al., 2017; Momtazmanesh et al., 2019). This study was the first to focus on aggression-affected inpatients with ScZ with at least 2 weeks of antipsychotics discontinuation. Different from previous studies (Miller et al., 2011; de Witte et al., 2014; Momtazmanesh et al., 2019), our results from Bonferroni's multiple comparison tests demonstrated no difference in inflammation phenotype between inpatients with ScZ without aggression and healthy controls. The contradictory results may be attributed to differences in statistical analysis methods used and the specific enrolled participants without any aggression behaviors during the disease course, which further verifies the inconsistent conclusions regarding inflammatory phenotypes in ScZ (Momtazmanesh et al., 2019). In sharp contrast, dramatical elevations of CRP, IL-6, IL-1β, and TNF-α were not only observed in inpatients with ScZ with aggression, but the elevated cytokines also correlated positively to the severity of aggression measured by MOAS score that is partly similar to the previous reports (Petrikis et al., 2015; Zhang et al., 2017; Orsolini et al., 2018; Momtazmanesh et al., 2019; Fond et al., 2021). Li et al. demonstrated positive correlations between higher plasma IL-17 or TGF-β1 and severity of aggression in patients with ScZ (Li et al., 2016), however, we found slightly lower serum IL-17 and higher serum TGF-β1 in individuals with ScZ with aggression as compared with those without aggression. These findings indicate the need for additional research to confirm the role of IL-17 and TGF-β1 in aggression onset. Among the functional redundancies of IL-6, IL-1β, and TNF-α, these proinflammation mediators potentially drive aggression in a sophisticated and coordinated network. These data collectively suggest the contributory role of systemic proinflammation in the occurrence of aggression in ScZ.

Gastrointestinal source of proinflammation was unveiled in deficit ScZ, and the leaky gut was identified as one of the prerequisites for the inflammatory pathophysiology (Severance et al., 2012, 2016; Barber et al., 2019; Ciháková et al., 2019; Maes et al., 2019b). Transcellular integrity, paracellular adherens, and tight junctions are demonstrated universally to be the structural basis for maintaining normal intestinal permeability. Permeability-related biomarkers such as I-FABP and Claudin-3 present at high levels in peripheral blood can reliably reflect the occurrence of leaky gut as they are released into circulation by enterocytes when intestinal epitheliums are compromised (Barmeyer et al., 2017). The evidence that remarkable increases in serum levels of I-FABP and Claudin-3 only in cases with ScZ with aggression behaviors in this study indicates the possible role of increased intestinal permeability in the onset of proinflammation-driven aggression that has not been previously reported.

Correspondingly, the peripheral blood concentration of LPS was significantly higher in an aggression-affected group with ScZ as compared with the non-aggression. Translocating LPS links with an exacerbation of inflammation response (Panpetch et al., 2020) and the following correlation analysis also showed positive correlativity between the circulating concentrations of proinflammation mediators and LPS, and also LPS responded sCD14. Furthermore, serum levels of both the LPS and sCD14 were found to be related to specific aggression behaviors (aggression against objects or toward others) or psychotic symptoms (positive PANSS). As LPS-specific host response, sCD14 circulates at high levels in the serum and interacts with translocating LPS to stimulate antigen-presenting cells *via* toll-like receptor 4 (TLR4) signaling (Tsukamoto et al., 2018). Under bacteria or LPS challenge, vascular endothelial cells and perivascular mast cells have been reported to express abundant TLR4, thus, initiating the production of inflammation cytokines (Zeuke et al., 2002). On the other hand, decreased host EndoCAb in peripheral blood failed to bind and clear LPS from circulation, which ensures a high serum level of LPS for a long time and subsequently maintains systemic inflammation (Kyosiimire-Lugemwa et al., 2020). It is also worth noting that serum BactDNA loads in cases with ScZ with aggression may have little effect on inflammation state given the results from correlation analysis and differential expressions of BactDNA among cases with or without aggression. We can only speculate that serum BactDNA loads quantified by qPCR likely underestimate the presence of BactDNA within whole blood and corresponding perturbation of inflammation markers may be transient. Collectively, these findings emphasize the implication of translocating LPS as well as sCD14 in the systemic inflammation response, and thus, argue for the potential causative relationship between BT and onset of aggression in ScZ.

Largely due to the failures of interpersonal inference, to develop a proper theory of mind or in sensory attenuation, ScZ was identified as one of the emotion recognition disorders (Demekas et al., 2020). Emotion recognition has also been suggested to underlie aggression in individuals with ScZ (Acland et al., 2021); however, that may be decreased by elevated low-grade inflammation (Balter et al., 2018, 2021). In this study, evidence that systemic proinflammation potentially initiated by serum LPS correlated with aggression severity in inpatients with ScZ implies the possible contribution role of serum LPS to aggressive behaviors *via* emotion misrecognition that has important implications for integrated treatments of aggression.

Unfortunately, at least five limitations exist in our study. At first, a structured clinical interview to determine the clinical diagnosis of subjects was not performed. Second, only single samples from participants in a single center were obtained, within-subject verification of related biomarkers and replication procedures in larger study populations from multicenter are expected. Third, higher circulating BactDNA load was observed in NScZ-Ag group compared with the healthy group (Figure 3B), while correlation analysis between BactDNA and inflammation in NScZ-Ag group was not conducted as inflammation cytokines did not differ between the two groups (Figure 1), thereby perplexing the function of BactDNA in pathogenesis with ScZ. Furthermore, as with all case-controlled clinical studies, present data failed to adequately explain the causal relationship between BT-caused inflammation response and aggression in ScZ, related animal experiments are expected for ethical considerations. At last, the molecular mechanism by which translocating LPS promotes systemic inflammation and thus drives aggression remains to be further investigated.

## Conclusion

In conclusion, this study verifies mainly the presence of leaky gut-caused BT and further correlates BT-derived LPS and soluble CD14 to the severity of aggression possibly by driving proinflammation response in cases with ScZ with aggression. These observations collectively indicate that BT may be a novel anti-inflammation therapeutic target for aggression prophylaxis and improving disease outcomes in patients with ScZ with aggression against objects and others.

## Data Availability Statement

The raw data supporting the conclusions of this article will be made available by the authors, without undue reservation.

## Ethics Statement

The studies involving human participants were reviewed and approved by the Ethics Committee of the Second People's Hospital of Zhumadian. The patients/participants provided their written informed consent to participate in this study.

## Author Contributions

CW and TZ: contributed equally to the manuscript. J-YF and B-TC: methodology, supervision, visualization, and writing—review and editing. CW and H-XD: clinical assessment, data curation, investigation, and writing—original draft. LH and X-LX: formal analysis and statistical analysis. All authors have contributed to and have approved the final manuscript.

## Funding

This work was funded by the grant from the National Natural Science Foundation of China (82003337) and China Postdoctoral Science Foundation (2020M683268).

## Conflict of Interest

The authors declare that the research was conducted in the absence of any commercial or financial relationships that could be construed as a potential conflict of interest.

## Publisher's Note

All claims expressed in this article are solely those of the authors and do not necessarily represent those of their affiliated organizations, or those of the publisher, the editors and the reviewers. Any product that may be evaluated in this article, or claim that may be made by its manufacturer, is not guaranteed or endorsed by the publisher.
